# Motivation for and adherence to growth hormone replacement therapy in adults with hypopituitarism: the patients‘ perspective

**DOI:** 10.1007/s11102-020-01046-y

**Published:** 2020-05-21

**Authors:** Ilonka Kreitschmann-Andermahr, Sonja Siegel, Nicole Unger, Christine Streetz-van der Werf, Wolfram Karges, Katharina Schilbach, Bernadette Schröder, Janine Szybowicz, Janina Sauerwald, Kathrin Zopf, Agnieszka Grzywotz, Martin Bidlingmaier, Heide Sommer, Christian J. Strasburger

**Affiliations:** 1 Department of Neurosurgery and Spine Surgery, University of Duisburg-Essen, Hufelandstrasse 55, 45147 Essen, Germany; 2 Department of Endocrinology, Diabetology and Metabolism, University of Duisburg-Essen, Hufelandstr. 55, 45147 Essen, Germany; 3 Division of Endocrinology and Diabetology, RWTH Aachen University Hospital, Pauwelsstrasse 30, 52074 Aachen, Germany; 4 Medizinische Klinik und Poliklinik IV, Klinikum der Universität München, Munich, Germany; 5 Hexal AG, Industriestrasse 25, 83607 Holzkirchen, Germany; 6 Department of Endocrinology, Diabetes and Nutritional Medicine, Campus Charité Mitte, Charité Universitaetsmedizin, Charitéplatz 1, 10117 Berlin, Germany

**Keywords:** Growth hormone deficiency, Adherence, Patients’ perspective

## Abstract

**Introduction:**

While reasons for non-adherence in children requiring growth hormone (GH) replacement (GH-Rx) are well researched, few studies have investigated adherence in adult GH deficient patients. Against the background of the adverse medical sequelae of untreated severe GH deficiency (GHD) in adults, we explored adherence to GH-Rx and associated factors in this patient group.

**Method:**

Cross-sectional analysis including 107 adult patients with severe GHD on GH-Rx, 15 untreated GDH patients and 19 who had discontinued therapy. Patients completed self-developed ad hoc surveys on adherence to medication and GH-Rx, specific beliefs about GH-Rx, side effects and burden of injection, reasons for never receiving or dropping out of therapy, respectively.

**Results:**

Adherence to GH-Rx was high (mean 15.8/18 points on the self-developed adherence score) and significantly correlated with general medication adherence. Higher age was significantly associated with better adherence to GH-Rx, while injection side effects, duration of treatment or device used were not. The most frequent reasons for not being on GH-Rx apart from medical reasons included fear of side effects, lack of belief in treatment effects and dislike of injections. In patients not on GH-Rx, the proportion of patients in employment was significantly smaller than in the treatment group, despite similar age and comorbidities.

**Conclusions:**

Adherence to GH-Rx was high for those patients on therapy. Instead of focusing on improving adherence in those adults already on GH-Rx, efforts should be undertaken to ally fear of side effects and provide education on positive treatment effects for those eligible but not receiving therapy.

**Electronic supplementary material:**

The online version of this article (10.1007/s11102-020-01046-y) contains supplementary material, which is available to authorized users.

## Introduction

Adherence has been defined by the World Health Organization as *the extent to which a person´s behavior - taking medication, following a diet, and/or executing lifestyle changes, corresponds with agreed recommendations from a health care provider* [1]. It is well known, that lack of adherence reduces the effects of therapies and negatively influences cost efficiency of medical interventions. Yet, according to the WHO’s 2003 report, adherence to medication in chronic diseases such as hypertension, diabetes mellitus and asthma was as low as 50% in industrialized countries at the time of publication, leading to a call for action [1].

Growth hormone (GH) deficiency (GHD) in adults (aGHD) is commonly caused by tumors of the sellar region (i.e. pituitary adenomas, craniopharyngeomas) and oftentimes associated with other comorbidities such as deficiency of other pituitary axes. GHD itself constitutes a chronic illness, associated with a high degree of adverse metabolic, cardiovascular and psychosocial sequelae [2–4]. Mortality in patients with pituitary disease including untreated GHD is increased as compared to the normal population [5]. Since the majority of the patients acquire the disease while in the middle of in their working lives, the associated productivity losses constitute an economic burden for society [6]. Nowadays, GHD is treated with recombinant human GH (rhGH) in children and adults, which was approved in the early 1990s for the adult indication after a number of studies proved positive effects of this replacement therapy on metabolic parameters and quality of life (QoL) [7–10]. GH replacement (GH-Rx) with rhGH constitutes a long-term therapy, requiring a daily subcutaneous injection. The effect of GH-Rx in children is easily appreciable by their change in linear growth. However, even though noticing beneficial treatment effects has an important influence on adherence, adherence to GH-Rx in children and adolescents is poor, leading to suboptimal growth in those missing numerous injections [11, 12]. A recent review showed that up to 71% of GH-deficient pediatric patients were non-adherent to their GH medication. Factors associated with non-adherence included long treatment duration and dissatisfaction with growth response, forgetting to administer the medication, poor administration techniques/convenience of the device used, lack of knowledge and understanding of the condition and treatment, the quality of the healthcare professional–patient relationship and sociodemographic factors, such as the level of parental school education [13]. For adults on GH-Rx, the literature on the topic is scarce. One study published in 2019 demonstrated that patients’ level of education and beliefs about therapeutic effect were strong drivers of treatment adherence [14]. Against the background of the adverse medical sequelae of untreated severe GHD in adults, we conducted the following cross-sectional questionnaire study in order to explore adherence to GH-Rx and associated factors in this patient group.

## Materials and methods

The study was conducted at one large German neurosurgical and four large endocrinological university referral centers (University of Essen-Duisburg, Depts. of Neurosurgery and Spine Surgery and Endocrinology, Diabetology and Metabolism; RWTH Aachen University, Dept. of Endocrinology; Charité University Berlin, Dept. Endocrinology; Klinikum der Universität München, Medizinische Klinik und Poliklinik IV), between June 2016 and September 2018. Medical records in the respective centers were screened for eligible adult patients (age between 21 and 80 years) with biochemically proven severe GHD. Additional patients were recruited via the regular endocrinological outpatient visits. Severe GHD had to be proven either by means of a GH stimulation test performed according to local standards with local cut-offs or by insulin-like growth factor-I (IGF-I) levels more than two standard deviation scores (SDS) below normal in the presence of proven deficiency of other pituitary axes [15]. Patients with known active psychotic illnesses and known insufficient fluency of the German language were excluded from participation. Patients were grouped into one of three groups: Patients on hrGH replacement at the time of the study, in the following called *treatment group*, patients, who had stopped rhGH-Rx at the time of the study (*drop-out group*), and patients who had never received rhGH-Rx despite proven GHD (*untreated group*).

Since validated questionnaires assessing adherence to GH-Rx were not available at the time of planning the study, we developed three ad hoc questionnaires in order to assess sociodemographic data, general adherence to medication and GH-Rx-specific adherence, based on a systematic literature research and our own experience in conducting patient reported outcome (PRO) research in neuroendocrinology. All patients received an information letter explaining the purpose of the study, a consent form and the three surveys which are described in more detail below. Medical data (diagnosis co-medication, treatments received due to pituitary disease etc.) were obtained from the case records of the respective centers.

### Design of the questionnaires

#### Questionnaire 1: sociodemographic data

The first questionnaire covered socio-demographic and medical data. It included questions on the level of education, employment situation, marital status, body height, weight, health insurance and previous therapies of the patients. Questionnaire 1 was handed out to all three patient groups.

#### Questionnaire 2: general adherence questionnaire

The second self-developed questionnaire focused on adherence with regard to medication in general. Items were chosen after reviewing the properties of general adherence scales available at the time of the study [16] [17], notably the Medication Adherence Questionnaire [18], the Self-efficacy for Appropriate Medication Use Scale [19], the Brief Medication Questionnaire [20] as well as the disease-specific Hill-Bone Compliance Scale [21] and the Medication Adherence Rating Scale [22]. From these, we constructed a general adherence scale with 6 items on how regularly and reliably patients take their medication (cf. Table 1 for the included items). The six questions were to be answered on a four-point Likert scale with values ranging between 0 and 3. The results from the six items were summed up to a General Adherence Score, which could range between 0 and 18 with higher values indicating a higher degree of adherence. Missing values on single scales were estimated by the mean value of the completed items. In addition to this scale, our questionnaire 2 also included 7 items outside the General Adherence Score. The additional questions referred to the patients’ knowledge of the reason for taking their medication and prescribed dose and frequency, their motivation to take the medication and helpful strategies to remind themselves to take the medication as well as the reasons to leave out the medication or reduce the medication. The questionnaire included yes/no items, multiple choice items and free text fields.

**Table 1:** Items of the General Adherence Questionnaire (GAQ) contributing to the Adherence Score

Item	Scale (0–3)
Do you always take your medication on the same time of the day?	Never/rarely/often/always
Did you forget to take your medication within the last 4 weeks?	Always/often/rarely/never
Do you sometimes forget your medication at home?	Always/often/rarely/never
How important for you is taking your medication regularly?	Unimportant/rather unimportant/ rather important/very important
Do you deliberately leave out your medication sometimes?	Always/often/rarely/never
Do you deliberately reduce the dose of your medication sometimes?	Always/often/rarely/never

#### Questionnaire 3

Three different versions of Questionnaire 3 were developed for the *treatment group* (Questionnaire 3a), the *drop-out group* (Questionnaire 3b) and the *untreated group* (Questionnaire 3c), in order to explore rhGH-specific adherence, reasons for stopping or declining rhGH replacement, respectively.

##### Questionnaire 3a: rhGH-specific adherence questionnaire

Questionnaire 3a focused on adherence specifically with regard to rhGH treatment. The questionnaire was filled in by the treatment group only. It included an rhGH-specific Adherence Score with 6 items. These items were nearly identical to those from the General Adherence Score (cf. Table 1). The phrase “medication” was replaced by “injection”, though, and patients were instructed to answer the questions with regard to their rhGH therapy. As with the General Adherence Score, the sum score of the six items could take values between 0 and 18. Additional items that did not count into the rhGH-specific Adherence Score focused on the reasons for rhGH therapy, details of the injection procedure, technical difficulties, symptoms after the injection, motivation to replace rhGH, helpful strategies to remind themselves of the injection and frequency of medical follow-ups. The questionnaire included yes/no items, multiple choice items and free text fields.

##### Questionnaire 3b: reasons for drop-out

Questionnaire 3b was handed out to the drop-out group only. It included 11 statements on possible reasons to terminate rhGH therapy. Patients were asked to which extent they agreed with these statements. Answers were given on a 5-point Likert-scale between “agree not at all” and “agree very much”. Additional reasons could be stated in a free text field.

##### Questionnaire 3c: reasons for declining therapy

Questionnaire 3c was filled in by the untreated group. It contained 9 items pertaining to possible reasons for declining rhGH therapy. Patients were asked to which extent they agreed with these statements. Answers were given on a 5-point Likert-scale between “agree not at all” and “agree very much”. Free text fields to state additional reasons and the physician’s advice concerning rhGH replacement were provided.

Additionally, a medical data sheet that covered medical information on diagnosis leading to GHD, therapies performed on the underlying illness (i.e. neurosurgery, radiotherapy), pituitary hormone replacement, comorbidities and comedication as well as current rhGH-Rx dose (where applicable), had to be filled out by the study physician of the respective center. The study protocol was approved by the local ethic committees of all participating centers with the lead vote provided by the Ethics Committee of the University of Duisburg Essen. Patients were included in the study if the signed consent form was returned with the filled-in questionnaire. The German version of the questionnaires as well as an English translation provided by the authors^1^ are available as supplementary material.

### Statistical analysis

**Fig. 1:**
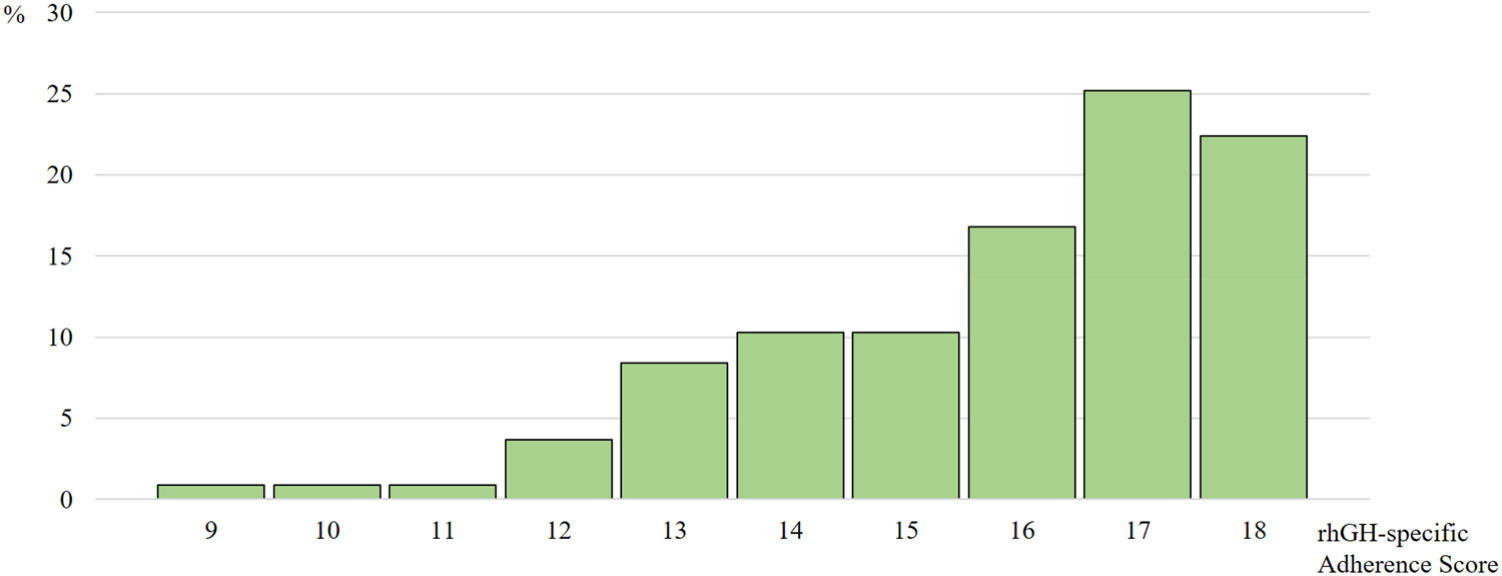
Distribution of the rhGH-specific adherence score

Database was generated by Microsoft Access 2010 (Microsoft Office 2010, Microsoft, Redmond/USA). Statistical analyses were conducted using IBM SPSS Statistics 22 (Statistical Package of the Social Sciences, SPSS Inc., Armonk/USA). Descriptive statistics of interval-scaled data were expressed as mean and standard deviations (SD), categorical data were expressed as absolute frequencies and valid percent (n, %). For the comparison of means between two groups student’s t-tests for unpaired variables were used. For more than two groups analyses of variance (ANOVA) were calculated, if variances were equal in the subgroups. If variances differed, the more robust Brown-Forsythe test was used. Nominal data were analyzed by chi-square test or, if expected frequencies were below 5, Fisher’s exact test. A visual screening of the histogram revealed a severe skewness of the rhGH-specific adherence score (cf. Fig. 1). Therefore, for correlation analyses including this score, the non-parametric Spearman’s Rho coefficient was used. For variables containing free text options (i.e. *Other reasons for leaving out an injection*), the answers were categorized and counted. Where applicable, a p-value of ≤ 0.05 was regarded as statistically significant.

## Results

### Study population

72 (51.1%) male and 69 (48.9%) female patients with a mean age of 51.0 ± 14.5 years at the time of the study participated. All of them suffered from severe GHD. The etiology of GHD included pituitary adenoma (n = 70), craniopharyngioma (n = 12), other tumors of the sellar and suprasellar region (n = 12, including 4 meningeomas, 3 pilocytic astrocytomas and 5 other pathologies), congenital (pan)hypopituitarism (n = 11; either genetic or due to hypothalamo-pituitary developmental lesions), empty sella syndrome (n = 6), idiopathic GHD (n = 6), cystic lesions of the pituitary (n = 4), hypophysitis (n = 4), mixed etiologies (such as trauma, Sheehan’s syndrome, congenital cerebral hemorrhage; n = 6). Data on GHD etiology was missing in n = 10 patients. 99 patients (71.2%) had undergone neurosurgery at any time during the disease and 35 patients (25.1%) had received radiation therapy of the pituitary region.

Next to severe somatotropic insufficiency, 84.4% of the study patients suffered from additional gonadotropic insufficiency (n = 119, 98 of them on substitution therapy) and 78.6% had thyreotropic insufficiency (n = 110, all of them substituted). In 77.1% (n = 108 patients) corticotropic insufficiency had been diagnosed. All but one of these patients required regular hydrocortisone replacement. 25.2% (n = 35) of the study patients also suffered from diabetes insipidus which necessitated antidiuretic hormone replacement in all cases. Diabetes mellitus was present in 9.6% (n = 11), hypertension in 40.9% (n = 47) and coronary heart disease in 3.5% of the patients (n = 4). 20.6% of the patients in the treatment group had started rhGH therapy during childhood (n = 21), while 79.4% had started therapy during adulthood (n = 81). 41.1% of the patients had a high educational level (*Fachabitur* = university of applied sciences entrance qualification, or higher, n = 58) and 54.6% of the patients were working full-time or part-time (n = 77) at the time of the study.

### Subgroups

107 patients (75.9%) currently received rhGH therapy (treatment group), 19 had stopped taking rhGH (13.5%, drop-out group) and 15 had declined therapy with rhGH (10.6%, untreated group). Sex distribution differed significantly between the three groups with 53.3% male patients in the treatment group, 21.1% male patients in the drop out group and 73.3% male patients in the untreated group (X^2^-test, p = 0.007) Mean age did not significantly differ between the subgroups (ANOVA, n.s.). Comorbidities were distributed equally in all three groups (X²-tests, n.s). The proportion of working patients differed significantly between the three groups. 61.7% of the patients in the treatment group were working, while only 46.7% of the untreated group and 21.1% of the drop-out group were working (X^2^-test, p = 0.004). Adherence to medication in general as assessed with our General Adherence Score was lowest in the drop-out group. The difference failed to reach significance, though. (Brown-Forsythe test, n = 0.148).

### Treatment group

#### rhGH administration

Of the 107 patients in the treatment group 99.1% (n = 106) injected themselves with rhGH, only one patient (0.9%) was helped by a partner. 93.5% used an injection pen and 6.5% (n = 7) used a pre-filled syringe. The average duration of rhGH therapy was 10.5 ± 9.5 years. Most patients (98.1%, n = 105) did not report any technical difficulties. One patient reported difficulties in remembering the right sequence of steps to start a new injection pen and one patient reported that a defective injection pen had to be replaced. The prescribed daily dose of rhGH was on average 0.34 ± 0.23 mg with a minimum of 0.05 mg and a maximum of 1.20 mg, with women on estrogen replacement requiring the highest doses (on average 0.51 ± 0.27 mg).

38.3% of the patients (n = 41) reported symptoms after the injection. Most common were bleeding (26.2%, n = 28), pain at the site of injection (19.6%, n = 21), bruising (8.4%, n = 9) and swelling (8.4%, n = 9). Other reported symptoms included skin irritation (4.7%, n = 5) and numbness or tingling of the hands (1.9%, n = 2). Of the 41 patients with symptoms after the injection, 51.2% (n = 21) felt they were not burdened by their symptoms at all, 24.4% (n = 10) were a bit burdened, 22% (n = 9) moderately burdened, 2.4% (n = 1) were considerably burdened and no single patient felt severely burdened by the injection-related symptoms.

#### Adherence

**Fig. 2:**
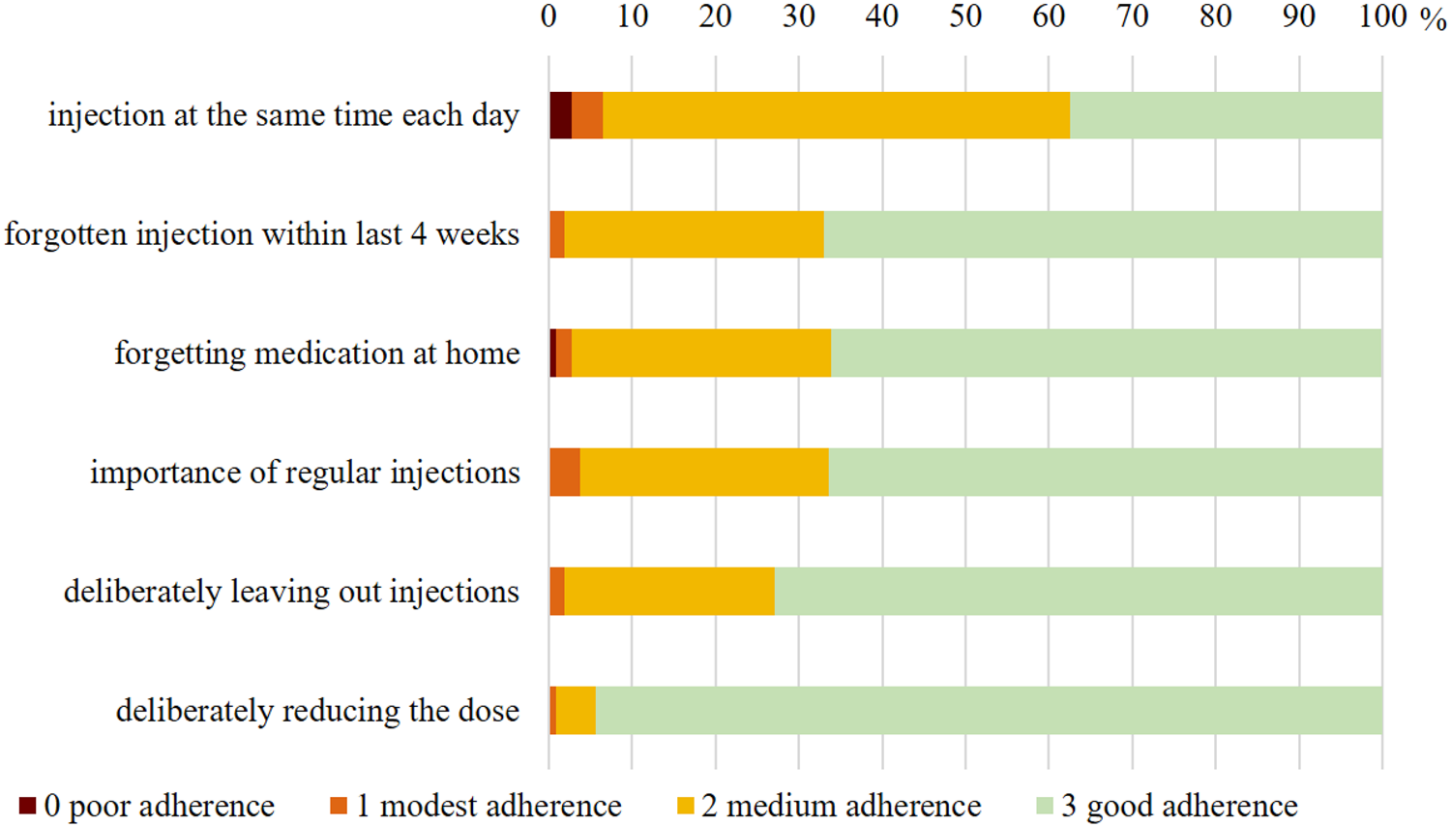
Relative frequencies of the answers to the rhGH-specific adherence questionnaire

The mean rhGH-specific Adherence Score in the treatment group was 15.8 ± 2.0 with a minimum of 9 and a maximum of 18. The distribution of adherence scores was severely skewed (cf. Fig. 1) 66.4% of the patients (n = 71) stated, that taking rhGH regularly was very important to them (cf. Fig. 2). 78.5% of the patients (n = 84) stated that their motivation to take rhGH was their physician’s advice to take it. Improving their physical capacity was the motivation for 63.6% (n = 68) and improving their mental capacity motivated 32.7% of the patients to replace rhGH (n = 35, multiple answers possible).

Only 37.4% of the patients (n = 40) reported to inject rhGH always at the same time of the day. 33% (n = 35) had forgotten to inject rhGH at least once within the last 4 weeks. 34% (n = 36) reported to forget their medication at home sometimes. 27.1% (n = 29) left out an injection deliberately sometimes. Reasons for leaving out an injection were being away from home (9,3%, n = 10), disliking the daily injection (9.3%, n = 10) and pain or discomfort during the injection (4.7%, n = 5). Only 1 patient (0.9%) stated to leave out injections due to side effects of the medication. Only 2 patients (1.9%) stated, that they felt so well that they thought they did not need the medication. None of the patients left out injections due to being unwell after the injection or due to technical difficulties. Only 5.6% (n = 6) reported to deliberately reduce the rhGH dose sometimes. Dose reduction was upon consultation with the treating physician in 3 cases, due to technical reasons (using up the rest of an injection pen) in 2 cases and due to discomfort during injections in 1 case.

#### Factors related to rhGH-specific adherence

rhGH-specific adherence was significantly related to general adherence to medication (Rho = 0.564, p = 0.000) and age (Rho = 0.223, p = 0.021), indicating that older patients adhered more to their rhGH treatment than younger ones. The duration of rhGH-therapy, the perceived burden through symptoms after the injection and the current rhGH-dose (n.s., cf. Table 2) were unrelated to rhGH-specific adherence. The mean rhGH-specific adherence score did not differ significantly between men (15.9 ± 2.0) and women (15.7 ± 2.1, t-test, n.s.). Educational level was unrelated to rhGH specific adherence (*Fachabitur* or higher 15.5 ± 2.1 vs. lower educational level 16.1.±1.9, t-test, n.s). Working patients had a significantly lower rhGH-specific adherence score (15.4 ± 2.0) than non-working patients (16.4 ± 2.0, t-test, p = 0.012). Childhood onset-GHD patients tended to be less adherent than adult-onset GHD patients (15.0 ± 2.8 vs.16.1 ± 1.7, t-test, n.s.), but the childhood onset group was also significantly younger than the adult-onset patient group (34.9 ± 10.0 years vs. 53.5 ± 12.4 years, t-test, p = 0.000).

**Table 2:** Factors related to rhGH-specific adherence as indicated by Spearman’s Rho

	Rho	P
General adherence to medication	0.564	0.000
Age	0.223	0.021
Perceived burden through symptoms after the injection	0.036	0.824
rhGH-dose	− 0.106	0.277
Duration of rhGH therapy	− 0.040	0.727

#### Drop-out group

**Fig. 3:**
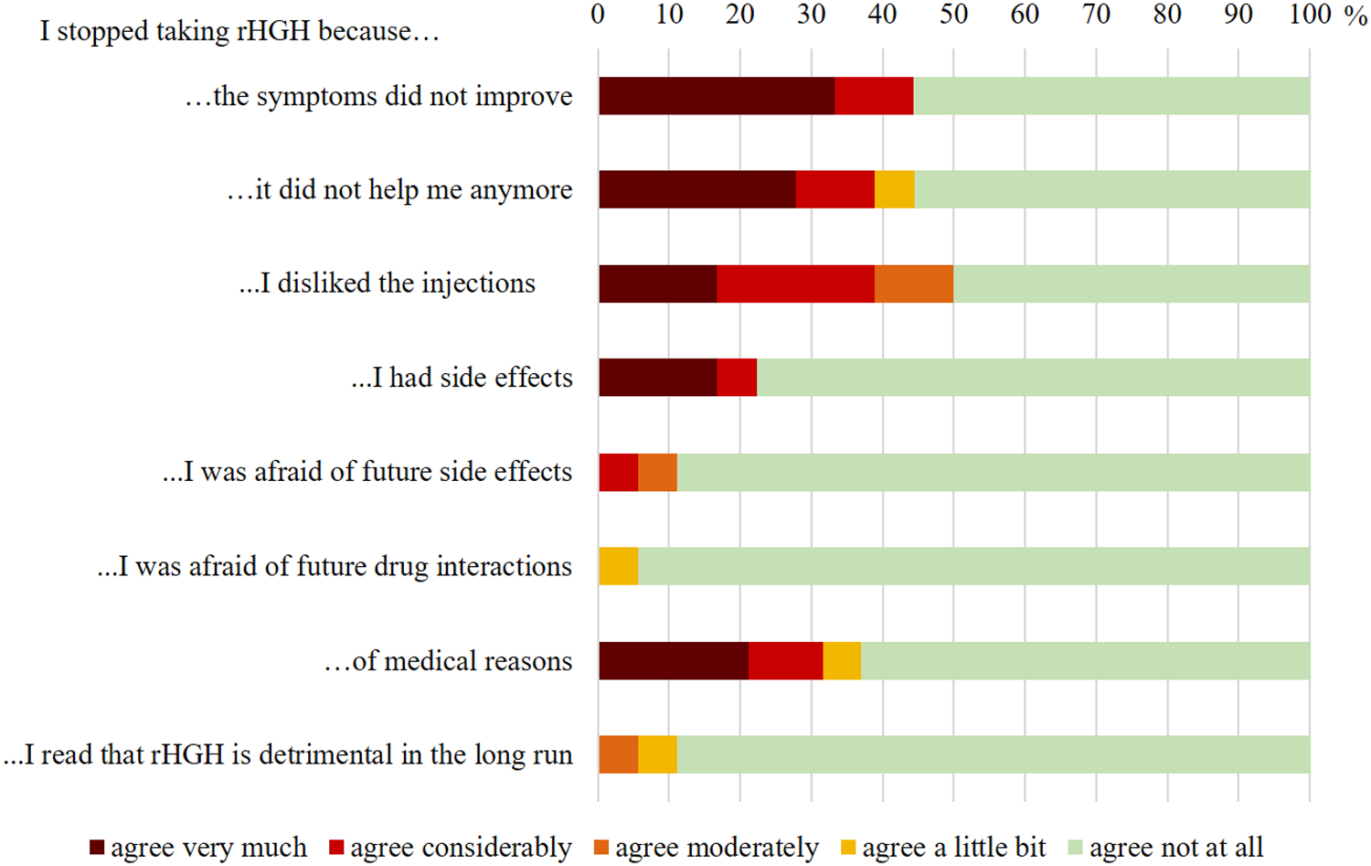
Reasons for drop out as stated in the questionnaire in relative frequencies

The average duration of rhGH replacement before drop out was 7.6 ± 8.1 years with a minimum of 1 year and a maximum of 27 years. Figure 3 shows the reasons for drop-out as stated in the questionnaire. Among the most common reasons were perceived lack of improvement, medical reasons and dislike of injections. Pregnancy, advice received from other patients against rhGH therapy and financial problems were not named as reasons for their decision by any patients (not depicted in Fig. 3).

### Untreated group

**Fig. 4:**
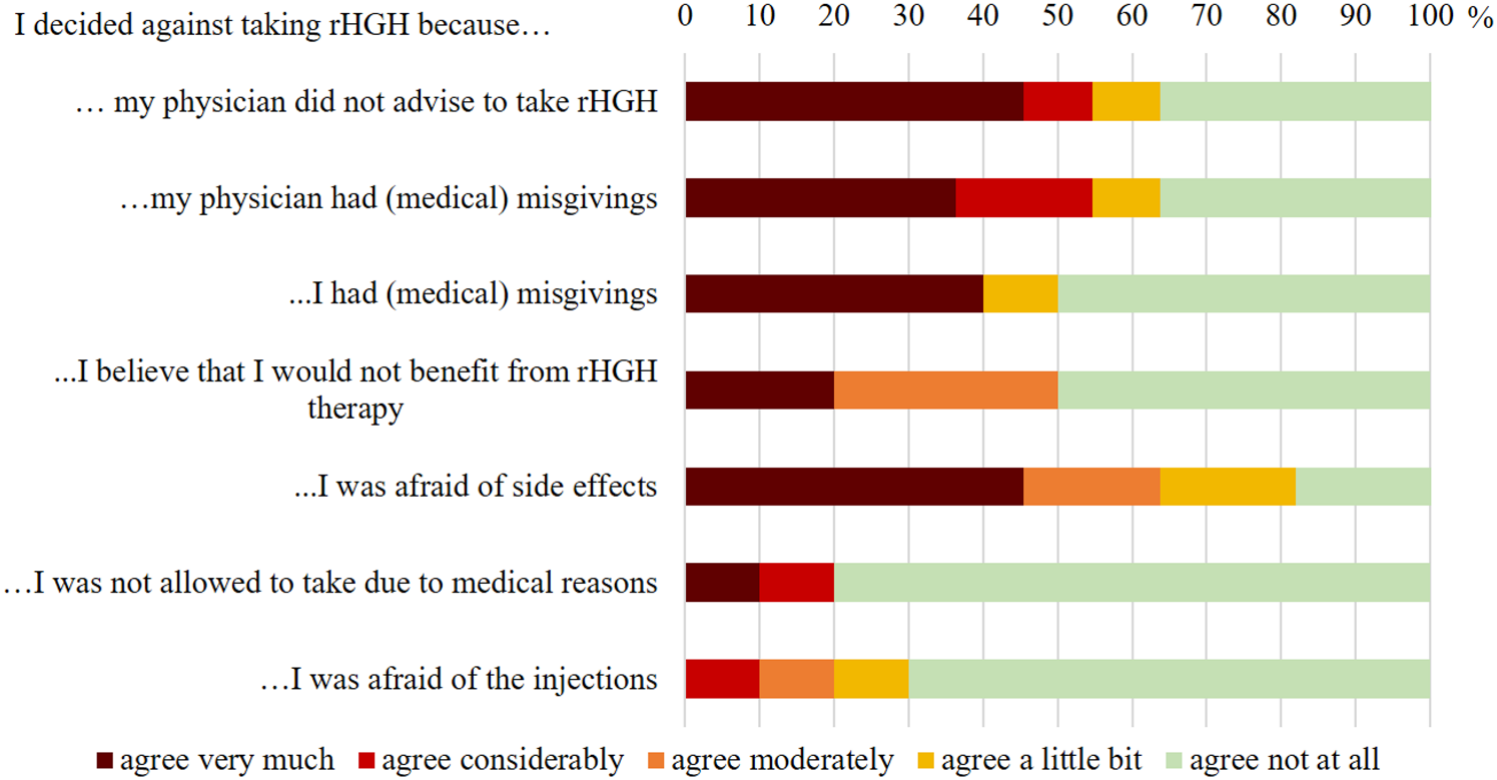
Reasons for declining rhGH therapy as stated in the questionnaire in relative frequencies

Of the 15 patients in the untreated group, 7 (46.7%) had been advised to take rhGH by their physician, 1 (7.7%) had not received any advice concerning rhGH and 7 (46.7%) had been advised against rhGH. Reasons for the physician’s advice against rhGH were risk of tumor growth (n = 5), old age (n = 1) and, at the time of the study, otherwise normal pituitary function (n = 1). Figure 4 shows the answers to the questionnaire on reasons for declining rhGH therapy in relative frequencies. The physician’s advice against rhGH therapy contributed considerably to the decision in more than half of the patients. Other major concerns were fear of side effects and lack of belief in a beneficial effect. None of the patients decided against therapy due to a wish for pregnancy or financial reasons (not depicted in Fig. 4).

## Discussion

In this study, we explored motivation for and adherence to rhGH replacement in a large cohort of patients with biochemically proven severe GHD. Approximately three quarters of our 141 patients (75.9%) currently received hrGH replacement while the rest either declined or dropped out of rhGH therapy, which is similar to the distribution of treated versus untreated patients in another recent smaller monocentric study on adherence to rhGH in adults by Amereller et al. [14].

Although almost 40% of our patients in our *treatment group* reported side effects of the injection, the majority had high rhGH specific adherence scores. Additionally, 67% of the patients reported not to have left out an injection within the last 4 weeks, again paralleling the results of the Amereller study, [14] in which 76% of 46 GHD patients on rhGH reported to never leave out an injection. This study, the present one and a 2016 publication by Auer et al. [23] who used prescription data to calculate adherence to rhGH demonstrate a much better degree of adherence in adult than in pediatric patients with severe GHD, in whom adherence is as low as 29% [13]. One reason for the higher degree of adherence to rhGH in adults may be found in the fact, that in contrast to pediatric GHD patients, side effects, administration techniques, handling of the injection device and duration of therapy were not related to non-adherence in our patient group. Interestingly, the level of adherence to rhGH in adult patients as identified in our study and the other two above-mentioned publications, is considerably higher than general adherence to medication in adult German patients as studied in a large cohort with more than 2500 participants [24]. In this representative sample of the German population, at least 33% of participants repeatedly failed to follow their doctor’s instructions and only 25% described themselves as fully adherent, with side effects of the medication being the strongest predictor of non-adherence. In contrast, 78.5% of patients in the *treatment group* reported here, relied on their physician’s advice as a reason for substituting rhGH and 66.4% stated that taking rhGH regularly was very important to them despite a high degree of reporting and feeling burdened by injection side effects. Since injection side effects played no role for adherence in our study and in view of the rather reserved attitude of GH-deficient adults toward switching to long-acting rhGH as reported in another study [14], we conclude that this form of therapy may play a less important role for adults than for children in improving adherence to rhGH replacement.

As in the study by Auer et al. [23], nonadherence to medication in our study was significantly more common in younger than in older patients, which is a finding also seen in the general German population [24] and may be explained by the assumption that older patients are more used to taking medication due to chronic conditions than younger individuals and rely more on medication for maintaining health. We also found overnight trips or being away from home in general to be frequent reasons for leaving out injections. Based on discussions with patients, we interpret this as a perceived limitation of lifestyle flexibility due to the inconvenience of taking the injection device along which is probably more bothersome for younger and more active than for older patients and may contribute to the explanation why also the group of working patients had a significantly lower rhGH specific adherence score than non-working patients.

Not surprisingly, financial concerns, which are major barriers to adherence in other health care systems [25, 26] were no reasons for poor adherence, discontinuing or never replacing rhGH in our cohort of adult patients with severe GHD, which is likely due to the fact that the vast majority of Germans have health insurances which impose no- or only insubstantial out-of-pocket contributions even for costly medications.

Sociodemographic status, as (parental) level of education, has been shown to be related to nonadherence in children and adults with GHD in other studies [13, 23]. In contrast, in the present investigation no effect of level of education or a significant difference in general adherence to medication was seen in the three investigated groups, leading to speculate that the decision against GH-Rx was an informed one, at least in some of the participants. However, as in other studies on the subject, perceived lack of efficacy and fear of side-effects were common reasons for drop-out or never substituting rhGH in our study. This accordance underscores the importance of informing patients of the long-term health benefits of rhGH replacement. Despite comparable age comorbidities, significantly less patients in the *untreated* and *drop-out groups* had employment relationships. This can be regarded as an indicator that GH-Rx provides long-term health benefits, enabling patients to participate in paid work.

While the large data set, the selection of patients with severe GHD due to serious organic damage to the pituitary and the comprehensive survey design constitute strengths of the study, there are also potential limitations. First, the use of subjective and retrospective measures of adherence which are prone to be biased by recall or social desirability effects, may have led to an overestimation of adherence in the *treatment group*. However, older studies have demonstrated that it is possible to effectively measure patient adherence using self-reports and interviews [27, 28]. We, therefore, do not consider the results to be seriously influenced by these factors. Yet, the asymmetry between the high number of patients on GH-Rx versus the smaller groups of 15 untreated patients and 19 dropped out of therapy could indicate a bias of analyzing preferentially a group of patients favorable to treatment. Secondly, although using a multicenter design, the proportion of patients never having received or having dropped out of rhGH therapy was rather small, and, therefore, limits the conclusions drawn, a restriction shared by the other survey study in the field.

In summary, we could show good adherence and a favorable attitude to rhGH replacement in adults which compares favorably to general adherence to medication in the German population and the 2003 WHO report [1, 24]. Next to the perceived positive treatment effects associated with the medication, this is possibly explained by the circumstance that in Germany rhGH replacement is mostly prescribed and monitored by large specialist practices or hospital outpatient departments with well-trained staff and a high degree of continuity. Instead of focusing on improving adherence in the adult patients already on rhGH replacement regimes, effort should be undertaken to ally fear of side effects and provide education on beneficial treatment effects for those eligible but not receiving therapy.

## Electronic supplementary material

Below is the link to the electronic supplementary material.

Supplementary material 1 (PDF 127.0 kb)

Supplementary material 2 (PDF 125.3 kb)

Supplementary material 3 (PDF 147.7 kb)

Supplementary material 4 (PDF 126.9 kb)

Supplementary material 5 (PDF 117.4 kb)

Supplementary material 6 (PDF 131.6 kb)

Supplementary material 7 (PDF 132.8 kb)

Supplementary material 8 (PDF 243.3 kb)

Supplementary material 9 (PDF 131.0 kb)

Supplementary material 10 (PDF 120.5 kb)

## Footnotes

1 Please note that this translation is intended to give an understanding of the questions asked but does not follow recommendations or guidelines for translating and validating a questionnaire.
