# Supplementary material for: Motivation for and adherence to growth hormone replacement therapy in adults with hypopituitarism: the patients‘ perspective
Source: Pituitary. 2020 May 21;23(5):479–87. doi: 10.1007/s11102-020-01046-y (PMC7426293; doi:10.1007/s11102-020-01046-y)
Supplement: Supplementary file 1 — Supplementary material 1 (PDF 127.0 kb) [file 11102_2020_1046_MOESM1_ESM.pdf]

Pituitary

Motivation for and Adherence to Growth Hormone Replacement Therapy in Adults with Hypopituitarism: The patients' perspective

Ilonka Kreitschmann-Andermahr, Sonja Siegel, Nicole Unger, Christine Streetz-van der Werf, Wolfram Karges, Katharina Schilbach, Bernadette Schröder, Janine Szybowicz, Janina Sauerwald, Kathrin Zopf, Agnieszka Grzywotz, Martin Bidlingmaier, Heide Sommer, Christian Joseph Strasburger
Corresponding Author: Ilonka Kreitschmann-Andermahr, University Hospital Essen, Germany; Ilonka.Kreitschmann@uk-essen.de

Patientenfragebogen I: Allgemeine Fragen zur Person und zum Wachstumshormonmangel

Liebe/r Patient/in,
im Folgenden finden Sie einige Fragen zu Ihrem Wachstumshormonmangel. Wir bitten Sie, alle Fragen vollständig zu beantworten und keine Fragen auszulassen.
Vielen Dank für Ihre Mitarbeit!

Persönliche Daten

ID-Code ☐

Heutiges Datum ☐

Alter ☐

Geschlecht
- ☐ männlich
- ☐ weiblich

Wohnort

Ort ☐

Postleitzahl ☐

Ausbildung

Höchster Schulabschluss:
- ☐ Keiner
- ☐ Hauptschulabschluss
- ☐ Realschulabschluss
- ☐ Fachabitur
- ☐ Abitur

Haben Sie eine abgeschlossene Ausbildung?
- ☐ Ja
- ☐ Nein

Haben Sie einen Hochschulabschluss?
- ☐ Ja
- ☐ Nein

AKTUELLE berufliche Situation

Beruf ☐

In Schichtarbeit?
- ☐ Ja
- ☐ Nein

Berufliche Situation
- ☐ Vollzeit
- ☐ Teilzeit
- ☐ Arbeitslos
- ☐ Hausfrau / Hausmann
- ☐ Berufsunfähigkeitsrente
- ☐ Frührente
- ☐ Rente

AKTUELLE Lebenssituation

Ich wohne:

- ☐ Alleine
- ☐ Alleine mit Kindern
- ☐ Mit meinem Lebenspartner
- ☐ Mit meinem Lebenspartner und Kindern
- ☐ In einer Wohngemeinschaft
- ☐ Bei meinen Eltern
- ☐ Betreut

Familienstand:

- ☐ Ledig
- ☐ Verheiratet
- ☐ Geschieden
- ☐ Verwitwet

Körperliche Daten

Größe (m)

Gewicht (kg)

Rauchen Sie? Wenn ja, wie viel?

☐ Ja, ________________ Zigaretten /Tag

☐ Nein

Krankenversicherung

Wie sind Sie krankenversichert?

- ☐ gesetzliche Krankenkasse
- ☐ private Krankenkasse
  - ☐ ohne Beihilfe
  - ☐ mit Beihilfe

Krankheitsverlauf

Aufgrund welcher Grunderkrankung haben Sie einen Wachstumshormonmangel?

Haben Sie, neben dem Wachstumshormonmangel, weitere Erkrankungen?

☐ Ja, ____

____

____

☐ Nein

Krankheitsverlauf

Wurden Sie jemals an der Hypophyse (Hirnanhangsdrüse) operiert?	Haben Sie jemals eine Strahlentherapie des Kopfes erhalten?
☐ Ja<br>☐ Nein	☐ Ja<br>☐ Nein
