# Supplementary material for: Motivation for and adherence to growth hormone replacement therapy in adults with hypopituitarism: the patients‘ perspective
Source: Pituitary. 2020 May 21;23(5):479–87. doi: 10.1007/s11102-020-01046-y (PMC7426293; doi:10.1007/s11102-020-01046-y)
Supplement: Supplementary file 2 — Supplementary material 2 (PDF 125.3 kb) [file 11102_2020_1046_MOESM2_ESM.pdf]

**Pituitary**

Motivation for and Adherence to Growth Hormone Replacement Therapy in Adults with Hypopituitarism: The patients‘ perspective

Ilonka Kreitschmann-Andermahr, Sonja Siegel, Nicole Unger, Christine Streetz-van der Werf, Wolfram Karges, Katharina Schilbach, Bernadette Schröder, Janine Szybowicz, Janina Sauerwald, Kathrin Zopf, Agnieszka Grzywotz, Martin Bidlingmaier, Heide Sommer, Christian Joseph Strasburger
Corresponding Author: Ilonka Kreitschmann-Andermahr, University Hospital Essen, Germany; Ilonka.Kreitschmann@uk-essen.de

# Patientenfragebogen II: Allgemeine Therapietreue

Liebe/r Patient/in,
wenn Sie (weitere) Medikamente nehmen, bitten wir Sie, die folgenden Fragen zu Ihrer medikamentösen Therapie zu beantworten.
Vielen Dank!

ID-Code ☐

Heutiges Datum ☐

**Kennen Sie den Grund für die Einnahme Ihrer Medikamente?**

☐ Ja

☐ Nein

**Wissen Sie, in welcher Dosis Sie Ihre Medikamente einnehmen müssen?**

☐ Ja

☐ Nein

**Wissen Sie, wie oft Sie Ihre Medikamente einnehmen müssen?**

☐ Ja

☐ Nein

**Was ist Ihre Motivation, die Medikamente einzunehmen?**

☐

**Nehmen Sie Ihre Medikamente immer zu gleichen Zeit?**
**(Bitte nur eine Antwort ankreuzen)**

☐ immer ☐ oft ☐ selten ☐ nie

**Haben Sie in den letzten 4 Wochen vergessen Ihre Medikamente zu nehmen?**
**(Bitte nur eine Antwort ankreuzen)**

☐ nie ☐ selten ☐ oft ☐ immer

**Vergessen Sie manchmal Ihre Medikamente zu Hause, wenn Sie unterwegs sind?**
**(Bitte nur eine Antwort ankreuzen)**

☐ nie ☐ selten ☐ oft ☐ immer

**Was hilft Ihnen dabei, sich an Ihre Medikamenteneinnahme zu erinnern?**

| |
|---|
| |

**Wie wichtig ist Ihnen die regelmäßige Einnahme Ihrer Medikamente?**
**(Bitte nur eine Antwort ankreuzen)**

☐ sehr wichtig ☐ eher wichtig ☐ eher unwichtig ☐ unwichtig

Warum?_________________________

**Lassen Sie manchmal absichtlich die Einnahme eines Medikaments aus?**
**(Bitte nur eine Antwort ankreuzen)**

☐ nie ☐ selten ☐ oft ☐ immer

**Wenn Sie die Einnahme auslassen, aus welchem Grund?**
**(Mehrfachnennungen möglich)**

☐ Weil ich mich nach der Medikamenteneinnahme manchmal schlecht fühle.
☐ Weil es mir so gut ging, dass ich das Medikament für unnötig hielt.
☐ Weil ich unangenehme Nebenwirkungen empfinde.
☐ Weil mir die Medikamenteneinnahme unangenehm ist.
☐ Weil ich Schwierigkeiten bei der Einnahme der Medikamente habe.
☐ Weil ich es lästig finde, so häufig Medikamente nehmen zu müssen.
☐ Sonstige Gründe: __________________________________

**Therapietreue - allgemein**

**Reduzieren Sie manchmal die Dosis Ihrer Medikamente?**
**(Bitte nur eine Antwort ankreuzen)**

☐ nie ☐ selten ☐ oft ☐ immer

**Wenn Sie die Dosis reduzieren, aus welchem Grund?**
**(Mehrfachnennungen möglich)**

☐ Weil ich mich nach der Medikamenteneinnahme manchmal schlecht fühle.

☐ Weil es mir so gut ging, dass ich die vorgesehene Dosis des Medikaments für unnötig hielt.

☐ Weil ich von der vorgesehenen Dosis unangenehme Nebenwirkungen habe.

☐ Weil mir die Medikamenteneinnahme unangenehm ist.

☐ Weil ich Schwierigkeiten bei der Einnahme der Medikamente habe.

☐ Weil ich es lästig finde, Medikamente nehmen zu müssen.

☐ Sonstige Gründe: ___________________________________
