# Supplementary material for: Motivation for and adherence to growth hormone replacement therapy in adults with hypopituitarism: the patients‘ perspective
Source: Pituitary. 2020 May 21;23(5):479–87. doi: 10.1007/s11102-020-01046-y (PMC7426293; doi:10.1007/s11102-020-01046-y)
Supplement: Supplementary file 3 — Supplementary material 3 (PDF 147.7 kb) [file 11102_2020_1046_MOESM3_ESM.pdf]

Pituitary

Motivation for and Adherence to Growth Hormone Replacement Therapy in Adults with Hypopituitarism: The patients‘ perspective

Ilonka Kreitschmann-Andermahr, Sonja Siegel, Nicole Unger, Christine Streetz-van der Werf, Wolfram Karges, Katharina Schilbach, Bernadette Schröder, Janine Szybowicz, Janina Sauerwald, Kathrin Zopf, Agnieszka Grzywotz, Martin Bidlingmaier, Heide Sommer, Christian Joseph Strasburger
Corresponding Author: Ilonka Kreitschmann-Andermahr, University Hospital Essen, Germany; Ilonka.Kreitschmann@uk-essen.de

Patientenfragebogen III a: Spezieller Fragebogen zum Wachstumshormonmangel

Liebe/r Patient/in,
im Folgenden finden Sie einige Fragen zu Ihrer Therapie mit Wachstumshormon. Wir bitten Sie, alle Fragen vollständig zu beantworten und keine Fragen auszulassen.
Vielen Dank für Ihre Mitarbeit!

Persönliche Daten

ID-Code ______	Heutiges Datum ______
Alter ______	Geschlecht<br>☐ männlich<br>☐ weiblich

Therapie mit Wachstumshormon

Aufgrund welcher Gründe hat Ihr Arzt Ihnen eine Behandlung mit Wachstumshormon empfohlen?

Was ist Ihre Motivation, Wachstumshormon (WH) zu spritzen? (Mehrfachnennungen möglich)

☐ Ich nehme WH, weil mein Arzt mir dazu rät.

☐ Ich nehme WH, weil sich meine körperliche Leistungsfähigkeit dadurch verbessert hat.

☐ Ich nehme WH, weil sich meine geistige Leistungsfähigkeit dadurch verbessert hat.

☐ Sonstiges: ______________________________

Therapie

Seit wann spritzen Sie Wachstumshormon?

	Jahr	Monat (wenn bekannt)
☐ Seit dem Kindesalter		
☐ Seit dem Erwachsenenalter (18 Jahre)		

Haben Sie jemals eine Pause der Therapie mit Wachstumshormon gemacht?

☐ Ja von ______ bis ______

☐ Nein

Wenn ja, aus welchem Grund?

______

In welcher Dosis spritzen Sie zur Zeit das Wachstumshormon?

______ mg/Tag

Spritzen Sie sich selbst?

☐ Ja

☐ Nein, eine andere Person spritzt mir das Wachstumshormon:

☐ Partner/in ☐ Hausarzt

☐ Freund/in ☐ Pflegedienst

☐ Angehörige/r ☐ Sonstige: ______

Wie wird das Wachstumshormon gespritzt?

☐ Pen ☐ Fertigspritzen zum Einmalgebrauch

Haben Sie technische Schwierigkeiten im Umgang mit dem Pen/der Einmalspritze?

☐ Ja, ______

______

______

☐ Nein

Therapie

Haben Sie nach der Injektion folgende Symptome? (Mehrfachantworten möglich)

☐ Schmerzen an der Injektionsstelle

☐ Nachblutungen

☐ Hautirritationen

☐ Dellen

☐ Sonstiges: ______________________________

☐ Ich habe keine Symptome nach dem Spritzen

Wie sehr belasten Sie diese Symptome? (Bitte nur eine Antwort ankreuzen)

☐ gar nicht ☐ wenig ☐ mittelmäßig ☐ ziemlich ☐ sehr

Wenn Sie aktuell noch zusätzliche Medikamente einnehmen, wie hoch sind die Kosten, die Sie selber durch Zuzahlungen zu tragen haben?

☐ Ca.____________________ Euro/Jahr Für das Wachstumshormon: ___________Euro/Jahr

☐ Ich weiß es nicht.

Therapietreue

Spritzen Sie immer zu gleichen Zeit? (Bitte nur eine Antwort ankreuzen)

☐ immer ☐ oft ☐ selten ☐ nie

Haben Sie in den letzten 4 Wochen vergessen sich zu spritzen? (Bitte nur eine Antwort ankreuzen)

☐ nie ☐ selten ☐ oft ☐ immer

Vergessen Sie manchmal Ihre Spritzen zu Hause, wenn Sie unterwegs sind? (Bitte nur eine Antwort ankreuzen)

☐ nie ☐ selten ☐ oft ☐ immer

Was hilft Ihnen sich daran zu erinnern, dass Sie sich spritzen müssen?

Therapietreue

Wie wichtig ist Ihnen das regelmäßige Spritzen des Wachstumshormons? (Bitte nur eine Antwort ankreuzen)

☐ sehr wichtig ☐ eher wichtig ☐ eher unwichtig ☐ unwichtig

Warum?____

Lassen Sie manchmal absichtlich eine Spritze aus? (Bitte nur eine Antwort ankreuzen)

☐ nie ☐ selten ☐ oft ☐ immer

Wenn Sie eine Spritze auslassen, aus welchem Grund? (Mehrfachnennungen möglich)

☐ Weil ich mich nach der Spritze manchmal schlecht fühle.
☐ Weil es mir so gut ging, dass ich die Spritze für unnötig hielt.
☐ Weil ich unangenehme Nebenwirkungen empfinde.
☐ Weil mir das Spritzen unangenehm ist.
☐ Weil ich technische Schwierigkeiten beim Spritzen habe.
☐ Weil ich es lästig finde, mich täglich spritzen zu müssen.
☐ Sonstige Gründe: ___________________________________

Reduzieren Sie manchmal die Dosis des Wachstumshormons? (Bitte nur eine Antwort ankreuzen)

☐ nie ☐ selten ☐ oft ☐ immer

Wenn Sie die Dosis reduzieren, aus welchem Grund? (Mehrfachnennungen möglich)

☐ Weil ich mich nach dem Spritzen manchmal schlecht fühle.
☐ Weil es mir so gut ging, dass ich die vorgesehene Dosis für unnötig hielt.
☐ Weil ich von der vorgesehenen Dosis unangenehme Nebenwirkungen habe.
☐ Weil mir das Spritzen unangenehm ist.
☐ Weil ich technische Schwierigkeiten beim Spritzen habe.
☐ Weil ich es lästig finde, mich täglich spritzen zu müssen.
☐ Sonstige Gründe: ___________________________________

Verlaufskontrolle der Therapie

Wie oft gehen Sie im Laufe eines Jahres aufgrund der Therapie mit Wachstumshormon zu Kontrolluntersuchungen?

☐

Zu welchem Arzt gehen Sie, um den Therapieverlauf mit Wachstumshormon kontrollieren zu lassen? (Mehrfachantworten möglich)

- ☐ Hausarzt
- ☐ Internist
- ☐ Endokrinologe
- ☐ Neurologe
- ☐ Neurochirurg
- ☐ Kardiologe
- ☐ Sonstige: ____________________

____

Haben Sie in Ihrem Zentrum/bei Ihrem Arzt immer den gleichen Ansprechpartner für Ihre Therapie mit Wachstumshormon?

☐ immer ☐ oft ☐ selten ☐ nie
