# Supplementary material for: Motivation for and adherence to growth hormone replacement therapy in adults with hypopituitarism: the patients‘ perspective
Source: Pituitary. 2020 May 21;23(5):479–87. doi: 10.1007/s11102-020-01046-y (PMC7426293; doi:10.1007/s11102-020-01046-y)
Supplement: Supplementary file 4 — Supplementary material 4 (PDF 126.9 kb) [file 11102_2020_1046_MOESM4_ESM.pdf]

Pituitary

Motivation for and Adherence to Growth Hormone Replacement Therapy in Adults with Hypopituitarism: The patients‘ perspective

Ilonka Kreitschmann-Andermahr, Sonja Siegel, Nicole Unger, Christine Streetz-van der Werf, Wolfram Karges, Katharina Schilbach, Bernadette Schröder, Janine Szybowicz, Janina Sauerwald, Kathrin Zopf, Agnieszka Grzywotz, Martin Bidlingmaier, Heide Sommer, Christian Joseph Strasburger
Corresponding Author: Ilonka Kreitschmann-Andermahr, University Hospital Essen, Germany; Ilonka.Kreitschmann@uk-essen.de

Patientenfragebogen III b: Spezieller Fragebogen zum Wachstumshormonmangel

Liebe/r Patient/in,
im Folgenden finden Sie einige Fragen zu Ihrer vorherigen Therapie mit Wachstumshormon. Wir bitten Sie, alle Fragen vollständig zu beantworten und keine Fragen auszulassen.
Vielen Dank für Ihre Mitarbeit!

Persönliche Daten

ID-Code ☐	Heutiges Datum ☐
Alter ☐	Geschlecht<br>☐ männlich<br>☐ weiblich

Therapieverlauf

Wenn Sie aktuell Medikamente einnehmen, wie hoch sind die Kosten, die Sie selber durch Zuzahlungen zu tragen haben?

☐ Ca.____________________ Euro/Jahr.

☐ Ich weiß es nicht.

Aufgrund welcher Gründe hatte Ihr Arzt Ihnen eine Behandlung mit Wachstumshormon empfohlen?

☐

Haben Sie zu Beginn der Therapie mit dem Wachstumshormon noch weitere Medikamente eingenommen? Wenn ja, welche?

☐ Ja, ____

____

____

☐ Nein

Therapieverlauf

Wann haben Sie die Therapie mit Wachstumshormon begonnen?

	Jahr	Monat (wenn bekannt)
☐ Im Kindesalter		
☐ Im Erwachsenenalter (18 Jahre)		

Wann haben Sie die Therapie mit Wachstumshormon beendet?

Jahr	Monat (wenn bekannt)

Auf der nächsten Seite geht es weiter!
Danke

Aus welchen Gründen haben Sie die Therapie mit Wachstumshormon beendet?

	Stimmt nicht	Stimmt wenig	Stimmt mittelmäßig	Stimmt ziemlich	Stimmt sehr
Das Medikament hat meine Symptome nicht wesentlich verbessert	☐	☐	☐	☐	☐
Das Medikament hat mir nicht mehr geholfen	☐	☐	☐	☐	☐
Die Injektionen haben mich gestört	☐	☐	☐	☐	☐
Ich hatte Nebenwirkungen	☐	☐	☐	☐	☐
Ich befürchtete Nebenwirkungen zu bekommen	☐	☐	☐	☐	☐
Ich befürchtete Wechselwirkungen mit anderen Medikamenten	☐	☐	☐	☐	☐
Ich musste die Behandlung aus medizinischen Gründen abbrechen	☐	☐	☐	☐	☐
Ich wurde schwanger	☐	☐	☐	☐	☐
Ich habe gelesen, dass das Medikament auf Dauer schlecht sei	☐	☐	☐	☐	☐
Andere Betroffene haben mir von dem Medikament abgeraten	☐	☐	☐	☐	☐
Die Zuzahlungen zu der Therapie waren mir zu hoch	☐	☐	☐	☐	☐

Sonstige Gründe:
