# Supplementary material for: Motivation for and adherence to growth hormone replacement therapy in adults with hypopituitarism: the patients‘ perspective
Source: Pituitary. 2020 May 21;23(5):479–87. doi: 10.1007/s11102-020-01046-y (PMC7426293; doi:10.1007/s11102-020-01046-y)
Supplement: Supplementary file 5 — Supplementary material 5 (PDF 117.4 kb) [file 11102_2020_1046_MOESM5_ESM.pdf]

Pituitary

Motivation for and Adherence to Growth Hormone Replacement Therapy in Adults with Hypopituitarism: The patients‘ perspective

Ilonka Kreitschmann-Andermahr, Sonja Siegel, Nicole Unger, Christine Streetz-van der Werf, Wolfram Karges, Katharina Schilbach, Bernadette Schröder, Janine Szybowicz, Janina Sauerwald, Kathrin Zopf, Agnieszka Grzywotz, Martin Bidlingmaier, Heide Sommer, Christian Joseph Strasburger
Corresponding Author: Ilonka Kreitschmann-Andermahr, University Hospital Essen, Germany; Ilonka.Kreitschmann@uk-essen.de

Patientenfragebogen III c: Spezieller Fragebogen zum Wachstumshormonmangel

Liebe/r Patient/in,
im Folgenden finden Sie einige Fragen zu Ihrem Wachstumshormonmangel. Wir bitten Sie, alle Fragen vollständig zu beantworten und keine Fragen auszulassen.
Vielen Dank für Ihre Mitarbeit!

Persönliche Daten

ID-Code ☐

Heutiges Datum ☐

Alter ☐

Geschlecht
- ☐ männlich
- ☐ weiblich

Wenn Sie aktuell Medikamente einnehmen, wie hoch sind die Kosten, die Sie selber durch Zuzahlungen zu tragen haben?

- ☐ Ca.____________________ Euro/Jahr.
- ☐ Ich weiß es nicht.

Wie hat Ihr Arzt Sie zur Therapie mit Wachstumshormon beraten?

- ☐ Mein Arzt hat mir die Therapie mit Wachstumshormon empfohlen.
- ☐ Mein Arzt hat mir von der Therapie mit Wachstumshormon abgeraten.
- ☐ Mein Arzt hat keine Empfehlung erwähnt.

Aufgrund welcher Gründe hat Ihr Arzt Ihnen eine Behandlung mit Wachstumshormon empfohlen oder davon abgeraten?

☐

Aus welchen Gründen haben Sie sich gegen eine Therapie mit Wachstumshormon entschieden?

	Stimmt nicht	Stimmt wenig	Stimmt mittelmäßig	Stimmt ziemlich	Stimmt sehr
Das Medikament wurde mir von meinem Arzt nicht empfohlen	☐	☐	☐	☐	☐
Mein Arzt hatte (medizinische) Bedenken in Bezug auf das Medikament	☐	☐	☐	☐	☐
Ich hatte (medizinische) Bedenken in Bezug auf das Medikament	☐	☐	☐	☐	☐
Ich glaube, die Therapie mit Wachstumshormon hat für mich keinen Nutzen	☐	☐	☐	☐	☐
Ich habe Bedenken wegen der Nebenwirkungen	☐	☐	☐	☐	☐
Ich hatte/habe Angst vor den Injektionen	☐	☐	☐	☐	☐
Ich war schwanger/wollte schwanger werden	☐	☐	☐	☐	☐
Ich durfte das Medikament aus anderen medizinischen Gründen nicht nehmen	☐	☐	☐	☐	☐
Die Zuzahlungen zu der Therapie waren mir zu hoch	☐	☐	☐	☐	☐

Sonstige Gründe:
