# Supplementary material for: Motivation for and adherence to growth hormone replacement therapy in adults with hypopituitarism: the patients‘ perspective
Source: Pituitary. 2020 May 21;23(5):479–87. doi: 10.1007/s11102-020-01046-y (PMC7426293; doi:10.1007/s11102-020-01046-y)
Supplement: Supplementary file 6 — Supplementary material 6 (PDF 131.6 kb) [file 11102_2020_1046_MOESM6_ESM.pdf]

Pituitary

Motivation for and Adherence to Growth Hormone Replacement Therapy in Adults with Hypopituitarism: The patients‘ perspective

Ilonka Kreitschmann-Andermahr, Sonja Siegel, Nicole Unger, Christine Streetz-van der Werf, Wolfram Karges, Katharina Schilbach, Bernadette Schröder, Janine Szybowicz, Janina Sauerwald, Kathrin Zopf, Agnieszka Grzywotz, Martin Bidlingmaier, Heide Sommer, Christian Joseph Strasburger

Corresponding Author: Ilonka Kreitschmann-Andermahr, University Hospital Essen, Germany; Ilonka.Kreitschmann@uk-essen.de

Patient Questionnaire I: General questions

Dear Patient,
Listed below, you will find some questions pertaining to yourself and your growth hormone deficiency. Please complete all questions fully and do not leave out any questions.
Thank you for your assistance!

Personal Data

ID-Code

Today‘s date

Age

Sex
- ☐ male
- ☐ female

Place of residence

Town

Postal code

Education

Highest school degree received:
- ☐ none
- ☐ basic secondary education
- ☐ secondary school certificate
- ☐ vocational baccalaureate
- ☐ university entrance certification

Have you completed a vocational training?
- ☐ Yes
- ☐ No

Do you have a university degree?
- ☐ Yes
- ☐ No

CURRENT professional situation

Profession

Do you work in shiftwork?
- ☐ Yes
- ☐ No

What is your current job situtation
- ☐ full time
- ☐ part time
- ☐ unemployed
- ☐ homemaker
- ☐ disability annuity
- ☐ early retirement
- ☐ retirement

CURRENT life situation

I live:

- ☐ alone
- ☐ alone with a child/children
- ☐ together with my partner
- ☐ together with my partner and children
- ☐ in a flat-sharing community
- ☐ with my parents
- ☐ in an assisted living

Marital status:

- ☐ single
- ☐ married
- ☐ divorced
- ☐ widowed

Physical data

Height (m)

Weight (kg)

Do you smoke, if yes how many cigarettes/day?

- ☐ Yes, ________________cigarettes /day
- ☐ No

Medical insurance

What kind of medical insurance do you have?

- ☐ social health insurance
- ☐ private health insurance
  - ☐ with state aids
  - ☐ without state aids

Course of the illness

What is the illness underlying your growth hormone deficiency?

Do you have any other diseases besides growth hormone deficiency ?

- ☐ Yes, ____
- ☐ No

Course of the illness

Did you have pituitary surgery?	Have you received radiotherapy to the brain?
☐ Yes<br>☐ No	☐ Yes<br>☐ No
