# Supplementary material for: Motivation for and adherence to growth hormone replacement therapy in adults with hypopituitarism: the patients‘ perspective
Source: Pituitary. 2020 May 21;23(5):479–87. doi: 10.1007/s11102-020-01046-y (PMC7426293; doi:10.1007/s11102-020-01046-y)
Supplement: Supplementary file 7 — Supplementary material 7 (PDF 132.8 kb) [file 11102_2020_1046_MOESM7_ESM.pdf]

**Pituitary**

Motivation for and Adherence to Growth Hormone Replacement Therapy in Adults with Hypopituitarism: The patients' perspective

Ilonka Kreitschmann-Andermahr, Sonja Siegel, Nicole Unger, Christine Streetz-van der Werf, Wolfram Karges, Katharina Schilbach, Bernadette Schröder, Janine Szybowicz, Janina Sauerwald, Kathrin Zopf, Agnieszka Grzywotz, Martin Bidlingmaier, Heide Sommer, Christian Joseph Strasburger
Corresponding Author: Ilonka Kreitschmann-Andermahr, University Hospital Essen, Germany; Ilonka.Kreitschmann@uk-essen.de

# Patient Questionaire II: General Adherence

Dear Patient, in case you take any medication next to growth hormone, please answer the following questions:

| ID-Code | Today's date |
|---|---|
| | |

**Do you know the reason for taking your medication?**

☐ Yes

☐ No

**Do you know in which dose you have to take your medication?**

☐ Yes

☐ No

**Do you know how often you have to take your medication?**

☐ Yes

☐ No

**What is your motivation for taking your medication?**

**Do you take your medication always at the same time?**
**(Please give only one answer)**

☐ always ☐ often ☐ rarely ☐ never

**During the last week, have you ever forgotten to take your medication?**
**(Please only mark one answer)**

☐ always ☐ often ☐ rarely ☐ never

**General adherecne**

**Do you sometimes forget your medication at home when you are underway?**
**(Please only mark one answer)**

☐ always ☐ often ☐ rarely ☐ never

**What helps you to remember to take your medication?**

| |
|---|
| |

**How important is taking your medication regularly to you?**
**(Please mark only one answer)**

☐ very important ☐ rather important ☐ rather unimportant ☐ not important at all

Why?_________________________

**Do you sometimes not take your medication deliberately?**
**(Please mark only one answer)**

☐ always ☐ often ☐ rarely ☐ never

**In case you sometimes leave out your medication deliberately, what is the reason?**
**(Multiple answers possible)**

☐ Because I sometimes feel bad after taking my medication.
☐ Because I felt so good, that I thought my medication was unnessessary.
☐ Because I had unpleasant side effects.
☐ Because taking medication makes me feel uncomfortable.
☐ Because I have difficulties taking my medication.
☐ Because I find it inconvenient to take medication so frequently.
☐ Other reasons: ___________________________________

## General adherence

**Do you sometimes reduce the dose of your medication?**
**(Please only mark one answer)**

☐ always ☐ often ☐ rarely ☐ never

**If you reduce the dose of your medication, why?**
**(Multiple answers possible)**

☐ Because I sometimes feel bad after taking my medication.

☐ Because I felt so good, that I thought the entire dose of my medication was unnessessary.

☐ Because I had unpleasant side effects with the prescribed dose.

☐ Because taking medication makes me feel uncomfortable.

☐ Because I have difficulties taking my medication.

☐ Because I find it inconvenient to take medication so frequently.

☐ Other reasons: ___________________________________
