# Supplementary material for: Motivation for and adherence to growth hormone replacement therapy in adults with hypopituitarism: the patients‘ perspective
Source: Pituitary. 2020 May 21;23(5):479–87. doi: 10.1007/s11102-020-01046-y (PMC7426293; doi:10.1007/s11102-020-01046-y)
Supplement: Supplementary file 8 — Supplementary material 8 (PDF 243.3 kb) [file 11102_2020_1046_MOESM8_ESM.pdf]

Pituitary

Motivation for and Adherence to Growth Hormone Replacement Therapy in Adults with Hypopituitarism: The patients‘ perspective

Ilonka Kreitschmann-Andermahr, Sonja Siegel, Nicole Unger, Christine Streetz-van der Werf, Wolfram Karges, Katharina Schilbach, Bernadette Schröder, Janine Szybowicz, Janina Sauerwald, Kathrin Zopf, Agnieszka Grzywotz, Martin Bidlingmaier, Heide Sommer, Christian Joseph Strasburger
Corresponding Author: Ilonka Kreitschmann-Andermahr, University Hospital Essen, Germany; Ilonka.Kreitschmann@uk-essen.de

Patient Questionnaire IIIa: Questions with regard to growth hormone deficiency

Dear patient,
In the following, you will find some questions about your therapy with growth hormone. Please answer all questions completely and do not leave out any questions. Thank you for your assistance.

Personal data

ID-Code ______	Today‘s date ______
Age ______	Sex<br>☐ male<br>☐ female

Therapy with growth hormone

For which reasons did your doctor recommend a therapy with growth hormone to you?

What is your motivation to self-inject growth hormone (GH)? (Multiple answers possible)

☐ I inject GH, because my doctor advises me to.

☐ I inject GH, because it improved my physical well-being.

☐ I inject GH, because it improved my mental well-being.

☐ other reasons: ______________________________

Therapy

Since when do you inject GH?

	year	month (if known)
☐ I started in childhood		
☐ I started in adulthood (> 18 years)		

Did you ever pause your GH therapy?

☐ Yes from ______ until ______

☐ No

If yes, what was the reason?

______

What is your daily GH dose?

______ mg/day

Do you self-inject?

☐ Yes

☐ No: I am being injected by:

☐ partner ☐ family physician

☐ friend ☐ mobile care service

☐ relative ☐ other: ______

What device do you use for injecting GH?

☐ pen ☐ prefilled syringes for single injection

Do you have technical difficulties in injecting GH?

☐ Yes, ______

______

______

☐ No

Therapy

Do you have one or more of the following symptoms after injecting GH (multiple answers possible)?

☐ pain at the injection site

☐ bleeding

☐ skin irritation

☐ swelling

☐ other: ________________________________

☐ I have no symptoms after the injection

How much do you feel burdend by your symptoms? (Please only mark one answer)

☐ not at all ☐ a little bit ☐ moderately ☐ considerably ☐ severly

If, at present, you are taking other medications, how much money do you have to pay for them yourself?

☐ ____________________ Euro/year For growth hormone: ___________Euro/year

☐ I don‘t know.

Adherence to GH therapy

Do you self-inject always at the same time? (Please only mark one answer)

☐ always ☐ often ☐ rarely ☐ never

Have you forgotten to self-inject within the last four weeks? (Please only mark one answer)

☐ always ☐ often ☐ rarely ☐ never

Do you sometimes forget your self-injection device when staying away from home? (Please only mark one answer)

☐ always ☐ often ☐ rarely ☐ never

What helps you to remember to inject GH?

Adherence to therapy

How important is injecting GH to you? (Please only mark one answer)

☐ very important ☐ rather important ☐ not so important ☐ unimportant

Why?____

Do you sometimes deliberately skip an injection? (Please only mark one answer)

☐ always ☐ often ☐ rarely ☐ never

If you skip an injection, what is the reason?
(Multiple answers possible)

☐ Because I sometimes feel bad after the injection.
☐ Because I felt so good that I thought that the injection was unnecessary.
☐ Because I had unpleasant side effects.
☐ Because I dislike injecting.
☐ Because I have technical difficulties with the injections.
☐ Because I think it is annoying to self-inject every day.
☐ Other reasons: ____________________________________

Do you sometimes reduce the dosis of the growth hormone?
(Please only mark one answer)

☐ always ☐ often ☐ rarely ☐ never

If you reduce the dose, for what reason?
(Multiple answers possible)

☐ Because I sometimes feel bad after the injection.
☐ Because I felt so good that I thought that the injection was unnecessary.
☐ Because I had unpleasant side effects.
☐ Because I dislike injecting.
☐ Because I have technical difficulties with the injections.
☐ Because I think it is annoying to self-inject every day.
☐ Other reasons: ____________________________________

Follow-up of therapy

Within the course of one year, how often do you have medical follow-up visits due to your growth hormone therapy?

[ ]

What kind of medical specialist do you see to have follow-up visits due to your growth hormone therapy? (Multiple answers possible)

- ☐ Family physician
- ☐ Internist
- ☐ Endocrinologist
- ☐ Neurologist
- ☐ Neurosurgeon
- ☐ Cardiologist
- ☐ Other: ____________________

In your medical center/in your medical practice, do you always have the same contact person for your growth hormone therapy?

☐ always ☐ often ☐ rarely ☐ never
