# Supplementary material for: Motivation for and adherence to growth hormone replacement therapy in adults with hypopituitarism: the patients‘ perspective
Source: Pituitary. 2020 May 21;23(5):479–87. doi: 10.1007/s11102-020-01046-y (PMC7426293; doi:10.1007/s11102-020-01046-y)
Supplement: Supplementary file 9 — Supplementary material 9 (PDF 131.0 kb) [file 11102_2020_1046_MOESM9_ESM.pdf]

Pituitary

Motivation for and Adherence to Growth Hormone Replacement Therapy in Adults with Hypopituitarism: The patients‘ perspective

Ilonka Kreitschmann-Andermahr, Sonja Siegel, Nicole Unger, Christine Streetz-van der Werf, Wolfram Karges, Katharina Schilbach, Bernadette Schröder, Janine Szybowicz, Janina Sauerwald, Kathrin Zopf, Agnieszka Grzywotz, Martin Bidlingmaier, Heide Sommer, Christian Joseph Strasburger

Corresponding Author: Ilonka Kreitschmann-Andermahr, University Hospital Essen, Germany; Ilonka.Kreitschmann@uk-essen.de

Patient questionnaire III b: Questions with regard to growth hormone deficiency

Dear patient,
In the following you will find some questions with regard to your prior therapy with growth hormone. Please answer all questions completely and do not leave out any questions. Thank you for your assistance!

Personal data

ID-Code

☐

Today‘s date

☐

Age

☐

Sex

☐ male

☐ female

Course of therapy

If you currently take any medication, how much do you pay for it yourself?

☐ ____________________ Euro/year.

☐ I don‘t know.

Why did your doctor recommend treatment with growth hormone to you?

☐

When commencing therapy with growth hormone, did you take any other medication?

☐ Yes, ______________________________

____

____

☐ No

Course of therapy

When did you start your therapy with growth hormone?

	Year	Month (if known)
☐ as a child		
☐ as an adult (> 18 years)		

When did you stop your therapy with growth hormone?

Year	Month (if known)

Please continue on the next page!

Thank you.

Why did you discontinue your therapy with growth hormone?

I agree	not at all	a little bit	somewhat	I rather agree	I fully agree
This medication did not improve my symptoms	☐	☐	☐	☐	☐
This medication did not help me any more	☐	☐	☐	☐	☐
I felt bothered by the injections	☐	☐	☐	☐	☐
I had side effects	☐	☐	☐	☐	☐
I was afraid to get side effects	☐	☐	☐	☐	☐
I was afraid of interactions with other medications	☐	☐	☐	☐	☐
I had to stop treatment for medical reasons	☐	☐	☐	☐	☐
I became pregnant	☐	☐	☐	☐	☐
I have read that the medication is bad for me in the long term	☐	☐	☐	☐	☐
Other patients have advised me not to take the medication	☐	☐	☐	☐	☐
The costs of therapy were too high for me	☐	☐	☐	☐	☐

Other reasons:
