# Supplementary material for: Motivation for and adherence to growth hormone replacement therapy in adults with hypopituitarism: the patients‘ perspective
Source: Pituitary. 2020 May 21;23(5):479–87. doi: 10.1007/s11102-020-01046-y (PMC7426293; doi:10.1007/s11102-020-01046-y)
Supplement: Supplementary file 10 — Supplementary material 10 (PDF 120.5 kb) [file 11102_2020_1046_MOESM10_ESM.pdf]

**Pituitary**

Motivation for and Adherence to Growth Hormone Replacement Therapy in Adults with Hypopituitarism: The patients‘ perspective

Ilonka Kreitschmann-Andermahr, Sonja Siegel, Nicole Unger, Christine Streetz-van der Werf, Wolfram Karges, Katharina Schilbach, Bernadette Schröder, Janine Szybowicz, Janina Sauerwald, Kathrin Zopf, Agnieszka Grzywotz, Martin Bidlingmaier, Heide Sommer, Christian Joseph Strasburger
Corresponding Author: Ilonka Kreitschmann-Andermahr, University Hospital Essen, Germany; Ilonka.Kreitschmann@uk-essen.de

# Patienten questionnaire III c: Questions with regard to growth hormone deficiency

Dear patient,
In the following you will find some questions with regard to your prior therapy with growth hormone. Please answer all questions completely and do not leave out any questions. Thank you for your assistance!

## Personal data

ID-Code

Today‘s date

Age

Sex

☐ male

☐ female

**If you currently take any medication, how much do you pay for it yourself?**

☐ ____________________ Euro/year.

☐ I don‘t know.

**How did your doctor advise you about growth hormone replacement therapy?**

☐ My doctor recommended growth hormone replacement to me.

☐ My doctor advised me against growth hormone replacement.

☐ My doctor did not give me any recommendation

**What were the reasons your doctor recommended or advised against growth hormone replacement?**

**Why did you decide against a therapy with growth hormone?**

| I agree | not at all | a litte bit | somewhat | I rather agree | I fully agree |
|---|---|---|---|---|---|
| This medication had not been recommended to me by my doctor | ☐ | ☐ | ☐ | ☐ | ☐ |
| My doctor had (medical) concerns about this medication | ☐ | ☐ | ☐ | ☐ | ☐ |
| I had (medical) concerns about this medication | ☐ | ☐ | ☐ | ☐ | ☐ |
| I thought that this medication was not helpful for me | ☐ | ☐ | ☐ | ☐ | ☐ |
| I had concerns about side effects | ☐ | ☐ | ☐ | ☐ | ☐ |
| I am/I was afraid of the injection | ☐ | ☐ | ☐ | ☐ | ☐ |
| I was pregnant / wanted to become pregnant | ☐ | ☐ | ☐ | ☐ | ☐ |
| I was not allowed to take this medication for medical reasons | ☐ | ☐ | ☐ | ☐ | ☐ |
| The out-of-pocket contributions for this therapy were too high for me | ☐ | ☐ | ☐ | ☐ | ☐ |

**Other reasons:**
